# Content validity for an autobiographical interview for older adults

**DOI:** 10.1590/2317-1782/20232022151es

**Published:** 2023-10-09

**Authors:** Pedro García Montenegro, Elenir Fedosse, Gabriel Urrutia Urrutia

**Affiliations:** 1 Departamento de Ciencias de la Fonoaudiología, Facultad de Ciencias de la Salud, Universidad de Talca - UTALCA - Talca (VII Región del Maule), Chile.; 2 Programa de Pós-graduação em Distúrbios da Comunicação Humana, Universidade Federal de Santa Maria - UFSM - Santa Maria (RS), Brasil.

**Keywords:** Autobiographical Memory, Autobiographical Interview, Aging, Future Time Perspective, Speech and Language Therapy/Logopedics

## Abstract

**Purpose:**

To validate a proposal for an autobiographical interview oriented to the typical older adult.

**Methods:**

Questions for a semi-structured autobiographical memory interview were designed and a protocol for its application was developed. Fourteen Speech and Language Pathologists judges and 14 older adults were used. Additionally, 2 interviews were conducted. Subsequently, content validity was obtained by means of Lawshe's classic procedure. Also, using a complementary evaluation for comprehensibility and length of the interview, as well as a data triangulation procedure with the judges and participants of the pilot experience.

**Results:**

Of the 22 items evaluated, only 4 were above the critical reference value (0.49).

**Conclusion:**

The need to incorporate this measure of analysis in the context of respect, identity and agency of older adults is discussed as part of a change in thinking under the gaze of person-centered care and a communicative competence model. As well as the need to incorporate different cultural paradigms and the use of digital technologies.

## INTRODUCTION

Every professional recognizes the importance of the interview and has no objections to categorizing it as a fundamental clinical tool when obtaining information and data from a patient^(1)^. Important reference works on the subject have been described, from medicine and psychology^(2)^, advancing on time in standardization processes to determine the degree of competence of the person who acts as an evaluator, as well as its importance for clinical decision-making^(3)^.

In this context and specifically for speech-language therapy, the generation of an effective and quality communication link is a fundamental premise for the clinician-person/patient relationship. The guidelines, organization, and structure of clinical interviews are quickly implemented as part of the most basic competencies of undergraduate training, incorporating various mechanisms to ensure the standardization and quality of this tool^(4)^, a fundamental element to comply with quality standards suggested by the evidence-based practice in speech-language therapy and that also highlights the dialogical nature of the relationship that this professional establishes with the beneficiaries of his services.

In general, clinical interviews have been hegemonized by guidelines of the biomedical model, focusing on the quantification of symptoms and signs specific to different possible pathological conditions, with little implementation of a specific tool based on the exploration of the phenomenological connotations that people attribute to the different autobiographical elements experienced throughout the life cycle and its impact on the present and future life situation.

Effective communication between clinician and patient is the basis to establish collaborative goals and negotiate relevant therapeutic objectives for all of them^(5)^. This starts with the interview process, the facilitations granted by the conversational tenor of an interview with an autobiographical orientation favor the naturalness of the personal story, the specific emphasis that each one gives to the facts/events, in which the phenomena become valid, interest and value in a unique way, a situation that traditional procedures cover since they are usually conducted under a questioning format.

In this context, an autobiographical interview collects potentially relevant information about situations, contexts, and experiences that the person has experienced. Although various researchers and clinicians have developed instruments based on these traditions, it is essential to differentiate the objectives pursued by each instrument, as well as their characteristics, orientations, and limitations^(6)^.

Although in recent years the use of narrative interviews has increased when exploring more phenomenological dimensions of the lives of people who assume the role of patients, their use continues to be infrequent, despite representing a positive way of reconstructing how current conditions they can be interpreted in the context of that person's life^(7)^. Consequently, the objective of this work was to validate a proposal for an Autobiographical Interview for older adults.

## METHODS

This research has a psychometric approach, assigned to a quantitative observational study, with a non-experimental cross-sectional design. Organizationally, it was structured in 4 stages: design, validation, preparation of the interview administration protocol, and its pilot application.

### Preparation of the autobiographical interview proposal

A semi-structured interview format was developed, based on theoretical references that consider life history as a significant axis for development^(8,9)^, maintaining attention on the interaction that takes place within the dialogic format. In particular, the elaboration of the instrument was adapted to the hermeneutics of interpretive phenomenological analysis^(10)^.

The original autobiographical interview consisted of a pool of 22 questions that were assigned to inquire about the experiences, 11 for life review and 11 for future prospecting.

### Validation of the content of the questions of the autobiographical interview

The designed questions were submitted for validation by a total of 28 judges, made up of a group of professional speech-language therapists (n=14; ten women and four men) and a group of older adults from the community (n=14, ten women and four men).

The inclusion criteria for the group of professionals were:

● To be certified speech-language therapists.

● To have at least five years of direct work experience with older adults, with a workload of no less than 11 hours; or

● To participate for at least five years in community volunteer activities - groups for older adults.

For the group of elderly judges, the inclusion criteria were:

● To be older than or equal to 60 years old.

● To have at least eight years of education (either continuous or discontinuous).

● To be enrolled in the primary health care unit of their community.

● Participate actively (at least one year old) in a community social group.

● To be retired from their work activities (retired).

● To have an auditory and visual acuity according to the requested activities or, failing that, to have the corresponding adaptations.

● To believe in a cognitive, functional, and emotional status within typical values:

- Score 1 on the Global Deterioration Scale - GDS^(11)^.
- Scores greater than or equal to 23 points on the Mini-mental State Examination - MMSE^(12)^.
- Scores less than or equal to 6 on the Pfeffer Functional Activities Questionnaire - PFAQ^(12)^.
- Scores less than or equal to 4 on the Goldberg General Health Questionnaire-12^(13)^.
- No medical history of cerebrovascular accident, brain injury, progressive neurological diseases - neurodegenerative and/or neuropsychiatric disorders.
- Without being in treatment with psychotropic drugs.

● To participate for at least three years in community volunteer activities and/or in groups for the elderly in the community.

To quantify the representativeness of the items proposed in the previous step, the content validity ratio (CVR) was calculated, according to the classic procedure described by Lawshe (1975), using a three-item evaluation scale, essential; useful but expendable; and unnecessary. The CVR is an indicator that is easy to calculate and interpret, being able to provide information both at the item level and the proposed instrument. The CVR oscillates between -1 and +1, with positive scores indicating greater representativeness of the items^(14)^.

### Preparation of the autobiographical interview protocol

After selecting the items with the procedure indicated above, an application protocol for the autobiographical interview was developed, as a circumscribed orientation for the interviewer. As it is a dialogical situation, the idea is to maintain the generic structure without falling into the stereotype of a clinical interview.

The protocol describes the execution of the interview in three phases^(1)^, contextualization of the process^(2)^; Exploration of the interview, with two subcomponents - (a) Exploration of the relevance of memories for the present - future; and (b) Exploration of the relevance of the future in the present and its prospecting - and^(3)^ Closing Process. An approximate time of 50 minutes - one hour was considered, protecting not to exceed the characteristic temporality of the cognitive stimulation activities that older adults receive in community centers.

The structuring of the interview protocol constitutes a guarantor of systematization to ensure its replicability^(15)^. This is intended to ensure coherence and organization when administering it since it is possible to verify that as the person progresses in the questions, a “natural deepening” of the information appears, from the appreciation of a general order to memories properly autobiographical. The interviewer must always maintain a role of neutrality and active listening, ruling out the formulation of judgments and intervening minimally, so as not to hinder or bias the interviewee's narrative.

### Interview phases

#### Contextualization of the interview process

It considers the opening of the interview, the execution of a lifeline as activities, which incorporate the reference to life stages and/or significant events in the life stages, and a contextualization question.

The opening to the subject initiates a framing, focusing on the figure of the elderly person in the present society, favoring the naturalness of the interaction by remaining permeable to dialogue and/or to different types of emerging comments.

The construction of the lifeline and its references to events were defined to reinforce the idea of continuity and direction of time, in addition to rescuing the framing that everyone defines and recognizes as relevant (life stages - life events). This allows us to appreciate the attribution of time that each person establishes for events - stages and even provides a form of estimation for what the person defines as “the rest of life,” which is particularly interesting when contrasting this proportion with the concrete estimate that people make of their future time in figures. This graph must always be available as a form of support or visual reinforcement during the interview.

#### Exploration phase of the interview

Although the positive valence of memories should always be emphasized, people evoke both positive and negative aspects in their stories, even going as far as to share them spontaneously. Although the literature reports this situation and since it is impossible to establish control of the affective valence of the memory^(16)^, it is necessary to respect the connotation given by the person and also reveal the constructive meaning of the experiences lived, arguing that the perspective of time allows us to see how a negative situation does not lose its condition of negativity, but has contributed to building values, resilience, foresight, self-care, among others, as the case may be.

It is not about *“turning a negative memory positive.”* The objective is to observe the memory in its context and with a temporal perspective. In any case, follow-up measures are incorporated during the closure that will be discussed later to identify and propose actions to manage any rumination behavior.

The phase of exploration - projection of memories begins by requesting the identification of the oldest memory. It is asked directly about the memory and narration is requested. Subsequently, the central questions of the interview are asked, emphasizing the greatest possible specificity, and inviting to deepen into the relevance that memories reflect in temporal contexts.

##### (a) Exploration of the relevance of memories in the present and future

To explore the relationship of reminiscences with both the present and the future, a visual analog scale from 1 to 10 is used as support (not at all relevant - very relevant - it changed my life). All information is coded for subsequent analysis^(17)^, according to the taxonomy of the classic Conway Memories model^(18)^:

- **Extended Memory:** They refer to extended periods of life that last much more than one day, such as childhood or school days.
- **Categorical Memory**: Actions that are repeated or similar event categories without specifying any specific moment (spatial or temporal), such as Christmas, birthdays, or summer vacations.
- **Specific Memory**: They describe specific moments that occur in a specific space and time and that can last from a few seconds to a few hours, such as an exam, the birth of a child, or a particular celebration. These memories are exceptionally longer than one day.
- **Memory generalization**: The inability to retrieve specific memories in the situations in which they are needed is called memory generalization. More than a memory, it is information of a semantic nature, value judgments, comments, or opinions regarding a situation, for example: “My childhood was very sad - I only have happy memories of my marriage, people from before were calmer.”

##### (b) Exploration of the relevance of the future in the present and its prospects

When deepening into the role of the future and its prospecting, reminiscences and previously made assessments are used as contextualization elements. It is requested to specifically quantify the future space of time that the interviewee visualizes, as well as an exploration that is as precise as possible and avoiding staying in comments of the type “whatever God wants,” “as long as my health lasts.” It is urged to deep into how relevant the value of past events (reminiscences) is in prospecting, which can be supported with the previously mentioned visual analog scale.

This phase of the interview is a space where the interviewer's interventions should be limited only to facilitating the detailed exploration of each memory.

#### Closing phase

In this phase, a self-evaluation of what the interviewee has reflected during the interview is facilitated. As the participation is more or less active, the process is encouraged, rescuing the fundamental concepts that the interviewee developed in the previous phase.

Since recalling certain life episodes or memories could generate some feelings of discomfort or emotionality in the interviewee, it is essential to give the interviewee a safe space and the opportunity to express themselves. The use of the telephone is specifically instructed in the event of any query or doubt regarding the feelings or emotions that may be experienced after the interview. The interviewee is informed at the time of consent, as well as two follow-up calls that are made after 48 hours and one week, respectively.

During the telephone contact, a brief questionnaire is applied, made up of questions that guide the general state of the interviewee. The results of the questions guide two decisions, visit to the center for specialized referral or follow-up control by the community program. It is always insisted that, in case of any doubt or consultation, the person can approach the clinical center during office hours or call by phone with their concerns and/or request an appointment.

#### Pilot application

To increase the validity of the results, a methodological triangulation was performed. Specifically, for the final proposal, in addition to considering the opinion and recommendations of the specialist and non-specialist judges, the interview was tested in a pilot application for a sample of 2 typical older adults, who met the inclusion criteria described in the section on non-specialist judges. It should be noted that each participant was duly informed and gave their consent.

A simple questionnaire was used as input to know the appreciation of the interviewees about the 4 questions and comments validated by the judges, through a Likert-type scaling and a section for comments. The comprehensibility and the length of the protocol were evaluated. The comprehensibility analysis considered the following categories: (i) clear and understandable, (ii) difficult to understand, and (iii) incomprehensible. Also, the extension considered an assessment of 5 degrees, from very extensive-strenuous, to very brief-insufficient.

### Ethical considerations

This Autobiographical Interview proposal was applied after the approval granted by the Universidade Federal de Santa María, Research Ethics Committee, Brasil, Folio No. 3.000.149. All participants also signed an informed consent form.

## RESULTS

### Content validity ratio

In this case, the critical value of the content validity ratio calculated based on the number of judges was 0.49^(19)^. Contrasting this index with the proposed questions, a structure composed of the 4 questions that exceeded the indicator was defined. These 4 questions were incorporated into the instrument, specifically in the interview phase (see Table 1).

**Table 1:** Definitive questions of the autobiographical interview proposal and its content validity ratio

Questions	CVR*
What meaning or value do you attribute to past events in the present moment?	0.73**
What meaning or value do you attribute to past events in the future?	0.6**
What meaning or value do you attribute to the future in the present moment?	0.73**
What is the most significant thing for you to imagine and project the future?	0.73**

* CVR: Content validity ratio;

** Significative CVR, greater than 0.49

Regarding the pilot study, the information valued by both participants is shown in Table 2. As we can see, it shows a positive reception of the instrument in general, highlighting that its extension, structure, and comprehensibility were relevant for the defined purpose, also welcoming the comments obtained.

**Table 2:** Analysis of the comprehensibility and extension of the interview protocol in the pilot application

	Participant 1		Participant 2	
	C^1^	E^2^	C	E
1. What meaning or value do you attribute to past events in the present moment?	Clear and understandable	Consistent extension	Clear and understandable	Somewhat extensive
2. What meaning or value do you attribute to past events in the future?	Clear and understandable	Consistent extension	Clear and understandable	Consistent extension
3. What meaning or value do you attribute to the future in the present moment?	Clear and understandable	Consistent extension	Clear and understandable	Consistent extension
4. What is the most significant thing for you to imagine and project the future?	Clear and understandable	Consistent extension	Clear and understandable	Consistent extension

C^1^= Understandable; E^2^= Extension

**Figure 1:**
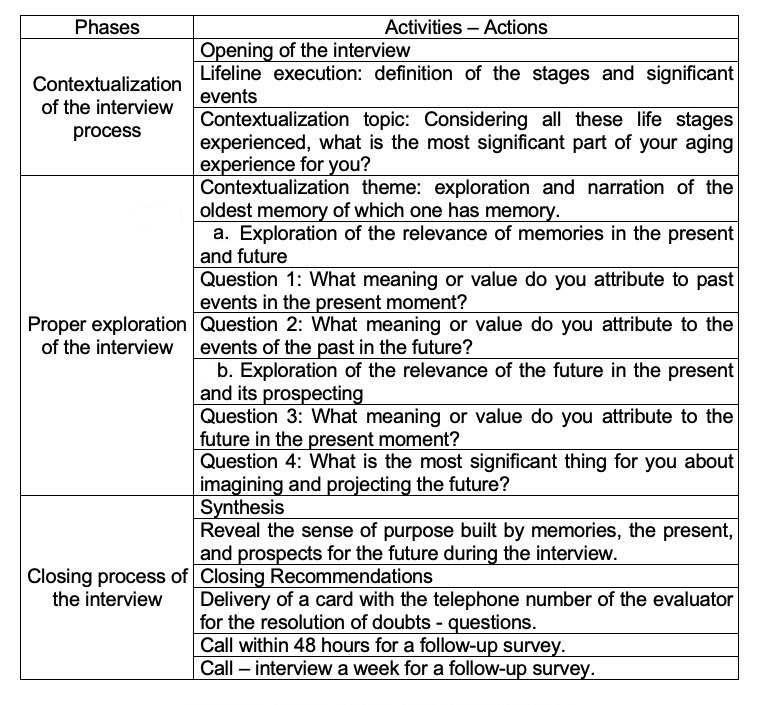
Autobiographical interview protocol

After the triangulation process, the final version of this interview proposal maintained the phases described in the protocol preparation stage, as shown in Figure 1.

It is important to emphasize that the recommendations for the interviewer are maintained and that it is also strongly suggested to obtain a digital record for the subsequent transcription of the content of the participant's narratives. Access to the final interview protocol is subject to contact with the principal investigator.

## DISCUSSION

There is a need to move towards reincorporating dialogue and the social construction of vital experiences in the measurements and procedures that clinicians use with the people they provide services^(20)^.

Although the advantage of having standardized interviews is recognized, it seems coherent that the proposed approach rescues the systematicity of the qualitative, as well as its exhaustiveness and parsimony. Authors such as Levine warn early and critically of the risks of laboratory evaluations and the problem of the lack of ecological validity of these evaluations. This decision is supported by five critical points:

1) From the most classical theory, Ricoeur (1991) argued that stories fragment experiences into smaller units, establishing beginnings, developments, and purposes that create a significant temporal landscape, a narrative goes beyond the chronology of the facts exposed since it is a way of conferring a sense of completeness on experiences^(21)^. Furthermore, the stories we tell ourselves not only build personal identities; they also configure a means of convergence to facilitate sociability; behavior that will be crucial for maintaining quality of life as we age. For speech-language therapists who work with older people, this information will facilitate interaction, allowing person-centered interventions to be configured, as is the case in some clinical contexts^(22)^.

2) The interest in personal narratives constitutes a vast opportunity framework for qualitative research in health sciences to develop and integrate social, clinical, and interpersonal contexts that are beyond quantifiable standards and values^(17)^. Also, the qualitative methodological tradition has been particularly enriched by interpretive phenomenological analysis (IPA), given its flexibility and orientation to the participant. The IPA offers the opportunity for the latter to commit to the story of the experiences lived, without being subject to restrictions or prosecutions. These participation formats permeate the dialogical interaction of the interview but also keep the narratives within the personal space of the person who remembers since he becomes the protagonist and agent of his story, a crucial relationship between memory and the perception of a development identity process, consistent and multidimensional^(23)^.

3) Unlike any other memory exploration task, the evaluations of its autobiographical component require an important and rigorous protocolization since by their nature they escape the control conditions that intervene in other types of evaluations.

The communality in all the autobiographical interviews is in requesting the narration of memory and its circumstantial elements, described in the most detailed way possible, emphasizing the quality of aspects such as its emotional valence, time-space coordinates, a perspective from which it is evoked (as stage, representation, or a mixture of both), among others. This also considers how complex it is to bring this very intimate and personal activity to the experimental paradigm that exists behind any laboratory condition^(6)^; the established stories are recreated interpretively, therefore, the logic of the facts passes to a paraphrase where there will be connotative decisions, of value, character, and depth along with many others before even delving into the most formal linguistic aspects of the organization of the discourse.

It is also relevant to remember that autobiographical stories represent a “travel in time”, that is, a displacement of consciousness towards another moment of the temporal continuum, and that in addition to performing its function (self-knowledge, managerial and social) it will materialize in an act of communication that will depend on specific rules for its co-construction, recursiveness, and form, as the models of communicative competence are understood^(24)^. In addition, given that the narratives are based on the phenomenology of the stated memories, it is plausible to deepen and directly overlap with the individual and manifest conceptions that the experiences of individuals reveal about themselves over time and about how these memories tell us about those who they are, of the construction of their identity and their state of subjective well-being.

4) Despite not being a unanimous fact, most researchers maintain that the narrative has a universal character; however, it is also recognized that narrative forms are culturally dependent; culture and temporal contexts generate interpretative frameworks that are unique^(25)^. The work of McAdams and others marks a transition between narrative conceptions focused on quasi-archetypal universals and studies of identities focused on the processes, contexts, and culture of individuals. It makes sense, then, to understand how social representations and stereotypes work, as well as to emphasize the different communicative and contextual aspects that occur in the interaction of the interview and the context of reminiscence as an activity. An example of this comes from the narratives of people with disabilities, where the events trace a process of acquiring a new identity.

5) It is worth noting the importance of increasing culturally diverse studies that detach from the Anglo-Saxon tradition. The experiences related to the use of personal narratives in socio-anthropological contexts are important, such as the proposals of Brazilian authors such as Ecléa Bosi^(26)^. This landmark work expands the reflection on procedures that validate the interrelationship and mutual influence between the researcher and the investigated individual, also emphasizing the profound need for an ethical and respectful act when inquiring into and with otherness, a motivation that has inspired us to procure the design of our instrument, rigorously complying with the methodological processes described for such purposes, reflecting since its creation the interest in the participation, leadership and their validation in the elderly people.

On the other hand, works such as that of Campo^(27)^ in Colombia, allowed us to reflect on the undeniable clinical contribution of structured instruments to exhaustively characterize the quantification of autobiographical events in the reports of normal typical populations of institutionalized and non-institutionalized older adults, during defined periods of life, establishing a counterpoint concerning the scope of less hegemonic qualitative approaches, such as the one in this proposal. Also, the phenomenological studies of Lolich^(28)^ in Argentina enrich the perspectives with other qualitative aspects, such as the use of an interpretive paradigm under the principles of the theory based on the narrative of events. There is still an interesting line of work that is relatively more recent, which has ventured into the exploration of digital channels and computerized social networks^(29)^, being a fact that the adult population, far from remaining static in the face of modern advances, is increasingly permeable to technology and the different digital resources as emerging media in the expression of identity.

Finally, it is recognized that establishing a communicative relationship of warmth and closeness generates a solid therapeutic bond, which facilitates deepening into the dimensions that affect the individual, which results as an adherence factor for this bond, allowing better management tools that impact people's quality of life. This study has limitations related to being more deeply embedded in the assessment of subjective well-being involved in the dialogical act of remembering, as well as not deepening more directly into elements of community participation and/or those of the relationship with other dimensions of interest in health, such as the experience of illness, mourning or physical pain^(30)^. The communality of autobiographical interviews comes from requesting narratives of memories contextualized by circumstantial elements, emphasizing the quality of aspects such as their emotional valence, the time-space coordinates, the perspective from which they are evoked (scenarios, representations, or a mixture of both), among others. This shows an important challenge for the discipline of speech-language therapy, interested in moving towards an appreciation of communication as a constitutive and integral element of the human being while recognizing the complexity of carrying out an activity as intimate and personal as remembering the paradigm that it exists behind any condition of traditional clinical care.

## CONCLUSION

Autobiographical interviews are positioned as tools of interest for professionals who work with elderly people, given the versatility and comprehensiveness that this instrument grants to exploration; Its clinical use may be limited, among others, due to lack of knowledge, apprehensions regarding its extension or fear of being overwhelmed by information, which is not traditionally oriented towards impediments and disabilities, which may lead the clinician to prefer to dismiss them. In this case, the facilitations granted by the conversational tenor of an interview with an autobiographical orientation favor the naturalness of the personal story, the specific emphasis that each one gives to the facts/events, in which the phenomena gain validity, interest, and value of a unique way, a situation that traditional procedures mask since they are usually conducted under an interrogation format.

However, there are professionals in Speech-language therapy who have been sufficiently visionary to build disciplinary knowledge based on the different contributions that the concepts of functionality, well-being, and participation have meant for the beneficiaries, along with a strong incorporation of a practice each time more evidence-based. This promising scenario allows the Autobiographical Interview to be an alternative that enables highly desirable clinical competencies such as the capacity for self-reflection and empathy in clinicians, as well as allowing our beneficiaries a truly comprehensive linguistic-communicative intervention, incorporating into the tradition of practice, the concern to offer scaffolding for the self-determination of identity, collective empowerment, the search for generativity, transcendence, and life purpose in the older age population.
