# Supplementary material for: Content validity for an autobiographical interview for older adults
Source: Codas. 2023 Oct 9;35(6):e20220151. doi: 10.1590/2317-1782/20232022151es (PMC10702701; doi:10.1590/2317-1782/20232022151es)
Supplement: Translation

# Translation: Validez de contenido para una entrevista autobiográfica para adultos mayores

**DOI:** 10.1590/2317-1782/20232022151pt

**Authors:** Pedro García Montenegro, Elenir Fedosse, Gabriel Urrutia Urrutia

**Affiliations:** 1 Departamento de Ciencias de la Fonoaudiología, Facultad de Ciencias de la Salud, Universidad de Talca - UTALCA - Talca (VII Región del Maule), Chile.; 2 Programa de Pós-graduação em Distúrbios da Comunicação Humana, Universidade Federal de Santa Maria - UFSM - Santa Maria (RS), Brasil.

**Keywords:** es: Memoria Autobiográfica, Entrevista Autobiográfica, Envejecimiento, Perspectiva de Tiempo Futuro, Fonoaudiología/Logopedia

## Abstract

**Objetivo:**

Validar una propuesta de Entrevista Autobiográfica orientada al adulto mayor típico.

**Método:**

Se diseñaron preguntas para una entrevista de memoria autobiográfica semiestructurada y se desarrolló un protocolo para la aplicación de esta. Se utilizaron 28 jueces; 14 fonoaudiólogos y 14 adultos mayores. Adicionalmente, se pilotaron 2 entrevistas. Posteriormente, la validez de contenido se obtuvo mediante el procedimiento clásico de Lawshe, utilizando complementariamente una evaluación para la comprensibilidad y extensión de la entrevista, así como un procedimiento de triangulación de datos con los jueces y participantes de la experiencia piloto.

**Resultados:**

De los 22 ítems evaluados, solo 4 estuvieron por sobre el valor crítico de referencia (0.49).

**Conclusión:**

Se discute sobre la necesidad de incorporar esta medida de análisis en el contexto del respeto, la identidad y la agencia de los adultos mayores, como parte de un cambio de paradigma bajo la mirada de las atenciones centradas en la persona y un modelo de competencia comunicativa. Así como la necesidad de incorporar paradigmas culturales diferentes y el uso de tecnologías digitales.

## INTRODUCCIÓN

Sin duda todo profesional reconoce la importancia de la entrevista y no tiene inconvenientes en categorizarla como una herramienta clínica fundamental al momento de obtener información y datos de un paciente^(1)^. Se han descrito importantes obras de referencia sobre el tema, principalmente desde la medicina y la psicología^(2)^ avanzando oportunamente en procesos de estandarización para la determinación del grado de competencia de quien actúa como evaluador, así como su importancia para la toma de decisiones clínicas^(3)^.

En este contexto y específicamente para la Fonoaudiología, la generación de un vínculo de comunicación efectivo y de calidad es una premisa fundamental para la relación clínico - persona/paciente. Los lineamientos, organización y estructura de las entrevistas clínicas son rápidamente implementados como parte de las competencias más básicas de la formación de pregrado, incorporado diversos mecanismos para asegurar la estandarización y calidad de esta herramienta^(4)^, elemento fundamental para cumplir con estándares de calidad sugeridos por la práctica basada en la evidencia en la actuación fonoaudiológica y que además releve el carácter dialógico de la relación que dicho profesional establece con los beneficiarios de sus prestaciones.

En general, las entrevistas clínicas han sido hegemonizadas por lineamientos del modelo biomédico, centrándose en la cuantificación de síntomas y signos propios a diferentes condiciones patológicas posibles, siendo escasa la implementación de una herramienta específica basada en la exploración de las connotaciones fenomenológicas que las personas atribuyen a los diferentes elementos autobiográficos vivenciados a lo largo del ciclo vital y su impacto en la situación de vida presente y futura.

Una comunicación efectiva entre clínico y paciente constituye la base sobre la que será factible establecer metas colaborativas y negociar objetivos terapéuticos relevantes para todos los interesados^(5)^. Esto parte con el proceso de entrevista: las facilitaciones otorgadas por el tenor conversacional de una entrevista con orientación autobiográfica favorecen la naturalidad del relato personal, el énfasis especifico que cada uno les da a los hechos/acontecimientos, con lo que los fenómenos cobran vigencia, interés y valor de un modo único, situación que los procedimientos tradicionales enmascaran, pues suelen ser conducidos bajo un formato de interrogación.

En este contexto, una entrevista autobiográfica recaba información potencialmente relevante sobre situaciones, contextos y experiencias que la persona ha vivenciado. Si bien varios investigadores y clínicos han desarrollado instrumentos basados en desde estas tradiciones, es fundamental diferenciar claramente los objetivos perseguidos por cada instrumento, así como sus características, orientaciones y limitaciones^(6)^.

Aunque en los últimos años ha aumentado el uso de entrevistas narrativas al momento de explorar dimensiones más fenomenológicas de la vida de las personas que asumen el rol de pacientes, su uso sigue siendo infrecuente, pese a representar una positiva forma de reconstruir cómo las condiciones actuales pueden ser interpretadas en el contexto de vida de esa persona^(7)^. En consecuencia, el objetivo de este trabajo consistió en validar una propuesta de Entrevista Autobiográfica para adultos mayores.

## MÉTODO

Esta investigación posee un enfoque psicométrico, inscribiéndose a un estudio observacional cuantitativo, con un diseño no experimental de corte transversal. Organizacionalmente, se estructuró en 4 etapas: diseño, validación, preparación del protocolo de administración de la entrevista y su aplicación piloto.

### Elaboración de la propuesta de entrevista autobiográfica

Se desarrolló un formato de entrevista semiestructurada, apoyándose en los referentes teóricos que consideran la historia de vida como eje significativo para el desarrollo^(8,9)^, manteniendo la atención sobre la interacción que se cumple dentro del formato dialógico. En particular, la elaboración del instrumento se adecuó a la hermenéutica del análisis fenomenológico interpretativo^(10)^.

La entrevista autobiográfica original constaba de un pool de 22 preguntas que se adscribían a inquirir sobre las experiencias; 11 para revisión de vida y 11 para la prospección de futuro.

### Validación del contenido de las preguntas de la entrevista autobiográfica

Las preguntas diseñadas fueron sometidas a validación por un total de 28 jueces, conformado por un grupo de profesionales fonoaudiólogos (n=14; diez mujeres y cuatro hombres) y un grupo de adultos mayores de la comunidad (n=14, diez mujeres y cuatro hombres).

Los criterios de inclusión para el grupo de profesionales fueron:

● Ser fonoaudiólogos titulados.

● Poseer al menos cinco años de experiencia de trabajo directo con personas mayores, con una carga horaria no menor a 11 horas; o

● Participar por al menos cinco años en actividades de voluntariado comunitarias - agrupaciones para personas mayores.

Para el grupo de jueces adultos mayores, los criterios de inclusión fueron:

● Tener una edad mayor o igual a 60 años;

● Contar con al menos ocho años de escolaridad (ya sea continua o discontinua);

● Estar inscrito en la unidad de atención primaria de salud de su comuna;

● Participar activamente (por lo menos con un año de antigüedad) en alguna agrupación social comunitaria;

● Encontrarse retirado de sus actividades laborales (jubilados);

● Poseer una acuidad auditiva y visual acorde a las actividades solicitadas o en su defecto, contar con las adaptaciones correspondientes;

● Acreditar un estatus cognitivo, funcional y emocional dentro de valores típicos:

- Puntaje 1 en la Global Deterioration Scale - GDS^(11)^.
- Puntuaciones mayores o iguales a 23 puntos en el Minimental State Examination - MMSE^(12)^.
- Puntuaciones menores o iguales a 6 en el Pfeffer Functional Activities Questionnaire - PFAQ^(12)^.
- Puntuaciones menores o iguales a 4 en el Cuestionario de Salud General de Goldberg-12^(13)^.
- Sin antecedentes médicos de: accidente cerebrovascular, traumatismo encéfalo-craneano, enfermedades neurológicas progresivas - neurodegenerativas y/o trastornos neuropsiquiátricos.
- Sin encontrarse en tratamiento con psicofármacos.

● Participar por al menos tres años en actividades de voluntariado comunitarias y/o en agrupaciones para personas mayores de la comunidad.

Para cuantificar la representatividad de los ítems propuestos en el paso anterior, se calculó la razón de validez de contenido (RVC), según el procedimiento clásico descrito por Lawshe (1975), empleando una escala de evaluación de tres elementos: esencial; útil pero prescindible; e innecesario. Se destaca que la RVC es un indicador fácil de calcular e interpretar, siendo capaz de aportar información tanto a nivel de ítem como del instrumento propuesto. La RVC oscila entre -1 y +1, siendo las puntuaciones positivas las que indican una mayor representatividad de los ítems^(14)^.

### Preparación del protocolo de entrevista autobiográfica

Después de seleccionar los ítems con el procedimiento indicado anteriormente, se desarrolló un protocolo de aplicación para la entrevista autobiográfica, como una orientación circunscrita para el entrevistador. Al tratarse de una situación dialógica, la idea es mantener la estructura genérica sin caer en el estereotipo de una entrevista clínica.

El protocolo describe la ejecución de la entrevista en tres fases: 1. contextualización del proceso; 2. Exploración propiamente tal de la entrevista, con dos subcomponentes: (a) Exploración de la relevancia de los recuerdos para el presente - futuro; y (b) Exploración de la relevancia del futuro en el presente y su prospección y 3. Proceso de Cierre. Se consideró un tiempo aproximado de 50 minutos - una hora, resguardando así, no superar la temporalidad característica de las actividades de estimulación cognitiva que reciben los adultos mayores en los centros comunitarios.

La estructuración del protocolo de la entrevista constituye un garante de sistematización a fin de asegurar su replicabilidad^(15)^. Con esto se pretende asegurar la coherencia y organización al momento de administrarla, puesto que es posible constatar que en la medida en que la persona avanza en las preguntas, aparece una “profundización natural” de la información, desde apreciaciones de orden general hacia los recuerdos propiamente autobiográficos. Debe destacarse además que el entrevistador ha de mantenerse siempre en un papel de neutralidad y escucha activa, descartando la formulación de juicios e interviniendo de modo mínimo, a fin de no entorpecer ni sesgar la narrativa del entrevistado.

### Fases de la entrevista

#### Contextualización del proceso de entrevista

Considera como actividades la apertura de la entrevista, la ejecución de una línea de vida, que incorpora la referencia a etapas de vida y/o a eventos significativos en las etapas de vida y una pregunta de contextualización.

La apertura al tema inicia un encuadre, enfocando a la figura del adulto mayor en la sociedad presente, favoreciendo la naturalidad de la interacción al mantenerse permeable al diálogo y/o a diferentes tipos de comentarios emergentes.

La construcción de la línea de vida y sus referencias a eventos se definió para reforzar la idea de continuidad y dirección del tiempo, además de rescatar el encuadre que cada individuo define y reconoce como relevante (etapas de vida - eventos de vida). Esto permite apreciar la atribución de tiempo que cada persona establece para los eventos - etapas e incluso, entrega una forma de estimación para lo que la persona define como “el resto de la vida”, lo que es particularmente interesante al contrastar dicha proporción con la estimación concreta que las personas hacen de su tiempo futuro en cifras. Esta gráfica ha de estar siempre disponible como forma de apoyo o reforzamiento visual durante la entrevista.

#### Fase de exploración propiamente tal de la entrevista

Cabe destacar que, aunque siempre se debe enfatizar en la valencia positiva de los recuerdos, las personas evocan tanto aspectos positivos como negativos en sus historias, llegando incluso a compartirlos espontáneamente. Aunque la literatura reporta esta situación y dado que es imposible establecer un control de la valencia afectiva del recuerdo^(16)^, es necesario respetar la connotación otorgada por la persona y además relevar el sentido constructivo de las experiencias vividas, aduciendo que la perspectiva del tiempo permite ver cómo una situación negativa no pierde su condición de negatividad, sino que ha aportado a construir valores, resiliencia, previsión, autocuidado, entre otros, según sea el caso.

Debe aclararse que no se trata de “volver positivo un recuerdo negativo”. El objetivo es observar el recuerdo en su contexto y con perspectiva temporal. De todos modos, se incorporan medidas de seguimiento durante el cierre que se discutirán más adelante, a fin de identificar y proponer acciones para manejar cualquier posible conducta de rumia.

En la fase de exploración - proyección de recuerdos se parte solicitando la identificación del recuerdo más antiguo del que se tiene memoria. Se pregunta directamente por dicho recuerdo y se solicita su narración. Posteriormente, se realizan las preguntas centrales de la entrevista, enfatizando en la mayor especificidad posible e invitando a adentrarse en la relevancia que reflejan los recuerdos en los contextos temporales.

##### (a) Exploración de la relevancia de los recuerdos en el presente y futuro

Para explorar la relación de las reminiscencias tanto con el presente como con el futuro se utiliza como apoyo una escala analógica visual de 1 a 10 (nada relevante - muy relevante - cambió mi vida). Toda la información es codificada para su posterior análisis^(17)^, según la taxonomía del modelo clásico de Recuerdos de Conway^(18)^, a saber:

- **Recuerdo Extendido:** refieren a períodos extensos de vida que duran mucho más de un día, como pueden ser la infancia o la época del colegio.
- **Recuerdo Categórico**: acciones que se repiten o a categorías de eventos similares sin especificar ningún momento concreto (espacial o temporal), como pueden ser las navidades, los cumpleaños o las vacaciones de verano.
- **Recuerdo Específico:** describen a momentos concretos que se dan en un espacio y tiempo específicos y que pueden durar desde unos segundos hasta unas horas, como un examen, el nacimiento de un hijo o una celebración en particular. Generalmente estos recuerdos excepcionalmente tienen una extensión mayor a un día.
- **Generalización del recuerdo:** la incapacidad para recuperar recuerdos específicos en las situaciones en las que se necesitan es lo que se llama generalización del recuerdo. Más que un recuerdo, se trata de información de carácter semántico, juicios de valor, comentarios u opiniones respecto a una situación, por ejemplo: “mi infancia fue muy triste - solo tengo recuerdos felices de mi matrimonio, la gente de antes era más tranquila”.

##### (b) Exploración de la relevancia del futuro en el presente y su prospección

Al adentrarse en el papel del futuro y su prospección, se utilizan como elementos de contextualización las reminiscencias y las valoraciones previamente hechas. Se solicita cuantificar específicamente el espacio de tiempo futuro que el entrevistado visualiza, así como una exploración lo más precisa posible y evitando quedarse en comentarios del tipo “lo que dios quiera”, “hasta donde me dure la salud”. Se insta a profundizar en cuan relevante es el valor de los eventos pasados (reminiscencias) en la prospección, lo que puede apoyarse con la escala analógica visual mencionada previamente.

Cabe destacar que esta fase de la entrevista es un espacio donde las intervenciones del entrevistador deben limitarse solo a facilitar la exploración detallada de cada recuerdo.

#### Fase de cierre

En esta fase se facilita una autoevaluación de que es lo que el entrevistado ha reflexionado de sí mismo durante la entrevista. En la medida que la participación es más o menos activa se incentiva el proceso, rescatando los conceptos fundamentales que el entrevistado desarrolló en la fase anterior.

Dado que rememorar ciertos episodios de vida o recuerdos podría generar algunos sentimientos de incomodidad o emocionalidad en el entrevistado es fundamental dar al entrevistado un espacio de seguridad y la oportunidad de expresarse. Se instruye específicamente en el uso del teléfono ante cualquier consulta o duda que se tenga respecto a los sentimientos o emociones que pudiese experimentar después de la entrevista. El entrevistado es informado al momento del consentimiento de esto, así como de dos llamadas de seguimiento que se realizan a las 48 horas y una semana respectivamente.

Durante el contacto telefónico se aplica un breve cuestionario conformado por preguntas que orientan sobre el estado general del entrevistado. Los resultados de las preguntas orientan dos decisiones: visita al centro para derivación especializada o seguimiento de control por parte del programa comunitario. Se insiste siempre en que, ante cualquier duda o consulta, la persona puede acercarse en horarios de oficina al centro clínico o llamar por teléfono por sus inquietudes y/o solicitar una cita.

#### Aplicación piloto

Con el fin de aumentar la validez de los resultados, se realizó una triangulación metodológica. Concretamente, para la propuesta definitiva además de considerar la opinión y recomendaciones de los jueces especialistas y no especialistas, la entrevista fue testeada en una aplicación piloto para una muestra de 2 adultos mayores típicos, los que cumplían con los criterios de inclusión descritos en el apartado de jueces no especialistas. Cabe destacar que cada participante fue debidamente informado y dio su consentimiento.

Se utilizó como insumo un cuestionario simple para conocer la apreciación de los entrevistados acerca de las 4 preguntas y comentarios validados por los jueces, mediante un escalamiento tipo Likert y un apartado para comentarios. Se evaluó la comprensibilidad por una parte y la extensión del protocolo por otra. El análisis de la comprensibilidad consideró las siguientes categorías: (i) clara y comprensible, (ii) difícil de entender y (iii) incomprensible. Por su parte, la extensión consideró una valoración de 5 grados, desde muy extensa-extenuante, hasta muy breve - insuficiente.

### Consideraciones éticas

Esta propuesta de Entrevista Autobiográfica se aplicó después de la aprobación otorgada por el Comité de Ética en Pesquisa de la Universidade Federal de Santa Maria, Brasil, Folio Nº 3.000.149. Todos los participantes firmaron además un formulario de consentimiento libre e informado.

## RESULTADOS

### Razón de validez de contenido

En este caso, el valor crítico de la razón de validez de contenido calculada en función del número de jueces fue de 0,49^(19)^. Contrastado dicho índice con las preguntas propuestas, se definió una estructura compuesta por las 4 preguntas que superaron tal indicador. Estas 4 preguntas fueron incorporadas al instrumento, específicamente en la fase de entrevista propiamente tal (ver Tabla 1).

**Tabla 1:** Preguntas definitivas de la propuesta de entrevista autobiográfica y su razón de validez de contenido

Pregunta	RVC*
¿Qué significado o valor le atribuye usted a los eventos del pasado en el momento presente?	0,73**
¿Qué significado o valor le atribuye usted a los eventos del pasado en el futuro?	0,6**
¿Qué significado o valor le atribuye usted al futuro en el momento presente?	0,73**
¿Qué es lo más significativo para usted de imaginar y proyectar el futuro?	0,73**

* RVC: Razón de validez de contenido;

** RVC significativo, mayor a 0,49

Con respecto al estudio piloto, la información valorada por ambos participantes se expone en la Tabla 2. Como se aprecia, apunta a una positiva recepción del instrumento en general, destacando que su extensión, estructura y comprensibilidad eran pertinentes para el propósito definido, acogiendo además los comentarios obtenidos.

**Tabla 2:** Análisis de la comprensibilidad y extensión del protocolo de entrevista en la aplicación piloto

	Participante 1		Participante 2	
	C^1^	E^2^	C	E
1. ¿Qué significado o valor le atribuye usted a los eventos del pasado en el momento presente?	Clara y comprensible	Extensión acorde	Clara y comprensible	Algo extensa
2. ¿Qué significado o valor le atribuye usted a los eventos del pasado en el futuro?	Clara y comprensible	Extensión acorde	Clara y comprensible	Extensión acorde
3. ¿Qué significado o valor le atribuye usted al futuro en el momento presente?	Clara y comprensible	Extensión acorde	Clara y comprensible	Extensión acorde
4. ¿Qué es lo más significativo para usted de imaginar y proyectar el futuro?	Clara y comprensible	Extensión acorde	Clara y comprensible	Extensión acorde

C^1^= Comprensibilidad; E^2^= Extensión

Es destacable que luego del proceso de triangulación, la versión definitiva de esta propuesta de entrevista mantuvo las fases descritas en la etapa de preparación de su protocolo, tal como se muestra en la Figura 1.

**Figura 1:** Protocolo de entrevista autobiográfica

Es pertinente enfatizar que las recomendaciones para el entrevistador se mantienen y que además se sugiere encarecidamente obtener un registro digital para la posterior transcripción del contenido de las narrativas de los participantes. El acceso al protocolo final de entrevista está sujeto al contacto con el investigador principal.

## DISCUSIÓN

Existe la necesidad de avanzar hacia una reincorporación del diálogo y la construcción social de las experiencias vitales en las mediciones y procedimientos que los clínicos utilizan con las personas a las que prestan servicios^(20)^.

Aunque se reconoce la ventaja que representa el contar con entrevistas estandarizadas, nos parece coherente que la aproximación propuesta rescate la sistematicidad de lo cualitativo, así como su exhaustividad y parsimonia. Autores como Levine, advierten temprana y críticamente de los riesgos de las evaluaciones de laboratorio y sobre la problemática de la falta de validez ecológica de estas evaluaciones. Esta decisión es sostenida por cinco puntos críticos:

1º) Desde la teoría más clásica, Ricoeur (1991) argumentaba que las historias fragmentan las experiencias en unidades menores, estableciendo inicios, desarrollos y finalidades que crean un paisaje temporal significativo, una narrativa va más allá de la cronología de los hechos expuestos, ya que se trata de una forma de conferirle sentido de completitud a las experiencias^(21)^. Además, las historias que nos contamos no solo construyen identidades personales; también configuran un medio de convergencia para facilitar la sociabilidad; conducta que será crucial para la mantención de la calidad de vida mientras envejecemos. Para los fonoaudiólogos que trabajan con personas envejecidas esta información facilitará la interacción, permitiendo configurar intervenciones centradas en la persona, como de hecho ocurre en algunos contextos clínicos^(22)^.

2º) El interés por las narrativas personales constituye un amplísimo marco de oportunidad para la investigación cualitativa en ciencias de la salud, a fin de desarrollar e integrar los contextos sociales, clínicos e interpersonales que están más allá de los estándares y valores cuantificables^(17)^. Asimismo, la tradición metodológica cualitativa se ha visto particularmente enriquecida con el análisis fenomenológico interpretativo (AFI), dada su flexibilidad y orientación al participante. En sí, el AFI ofrece la oportunidad de que este se comprometa con el relato de las experiencias vividas, sin ser sujeto de restricciones o enjuiciamientos. Estos formatos de participación permeabilizan la interacción dialógica de la entrevista, pero también mantienen las narrativas dentro del espacio personal del que rememora, pues es él quien se hace protagonista y agente de su historia, relación crucial entre el recuerdo y la percepción de un proceso desarrollo identitario consistente y multidimensional^(23)^.

3º) A diferencia de cualquier otra tarea de exploración de la memoria, las evaluaciones de su componente autobiográfico requieren de una importante y rigurosa protocolización, puesto que por su naturaleza escapan ampliamente de las condiciones de control que intervienen en otro tipo de evaluaciones.

La comunalidad en todas las entrevistas autobiográficas está en solicitar la narración de un recuerdo y sus elementos circunstanciales, descritos del modo más detallado posible, enfatizando en la calidad de aspectos tales como su valencia emocional, coordenadas de tiempo - espacio, perspectiva desde donde se evoca (como escenario, representación o una mezcla de ambos), entre otras. Esto también considera lo complejo que es llevar esta actividad tan íntima y personal al paradigma experimental que existe detrás de cualquier condición de laboratorio^(6)^; los relatos establecidos son recreados interpretativamente, por tanto, la lógica de los hechos pasa a una paráfrasis donde existirán decisiones connotativas, de valor, carácter y profundidad junto a muchas otras antes de siquiera adentrarse en los aspectos lingüísticos más formales de la organización del discurso.

También es relevante recordar que los relatos autobiográficos representan un “viaje en el tiempo”, es decir, un desplazamiento de la conciencia hacia otro momento del continuo temporal y que además de desempeñar una función propia (autoconocimiento, directivo y social) se materializará en un acto de comunicación que dependerá de reglas específicas para su co-construcción, recursividad y forma, como se comprenden los modelos de competencia comunicativa^(24)^. Además, dado que las narrativas se sustentan bajo la fenomenología de los recuerdos enunciados, es plausible profundizar e imbricarse directamente con las concepciones individuales y manifiestas que las experiencias de los individuos develan sobre sí mismos en el tiempo y sobre cómo dichos recuerdos nos hablan de quienes son, de la construcción de su identidad y de su estado de bienestar subjetivo.

4º) Pese a no ser un hecho completamente unánime, la mayoría de los investigadores sostiene que la narrativa posee un carácter universal; no obstante, también se reconoce que las formas narrativas son culturalmente dependientes; la cultura y los contextos temporales generan marcos interpretativos que son únicos^(25)^. El trabajo de McAdams y otros marca una transición entre las concepciones narrativas enfocadas en universales casi arquetípicos y los estudios de identidades focalizadas en los procesos, los contextos y la cultura de los individuos. Tiene sentido entonces comprender cómo actúan las representaciones y estereotipos sociales, así como recalcar en los diferentes aspectos comunicativos y contextuales que se dan en la interacción de la entrevista y en el contexto de la reminiscencia como actividad. Un ejemplo de esto proviene de las narrativas de personas en situación de discapacidad, donde los eventos trazan un proceso de adquisición de una nueva identidad.

5º) Cabe destacar la importancia de incrementar estudios culturalmente diversos, que se desapeguen de la tradición anglosajona. Son importantes las experiencias relacionadas con el uso de narrativas personales en contextos socio-antropológicos como las propuestas de autores brasileños como Ecléa Bosi^(26)^. Este trabajo señero, expande la reflexión sobre procedimientos que validen la interrelación y mutua influencia entre investigador e individuo investigado, enfatizando además en la profunda necesidad de un actuar ético y respetuoso al momento de indagar en y con las alteridades, motivación que nos ha inspirado al procurar el diseño de nuestro instrumento, cumpliendo rigurosamente con los procesos metodológicos descritos para tales fines, reflejando desde su creación el interés por la participación, protagonismo y validación de los mismos de los adultos mayores.

Por otra parte, trabajos como el de Campo^(27)^ en Colombia, permitieron reflexionar sobre el innegable aporte clínico de instrumentos estructurados para caracterizar exhaustivamente la cuantificación de eventos autobiográficos en los relatos de poblaciones normales o típicas de adultos mayores institucionalizados y no institucionalizados, durante periodos definidos de vida, estableciendo un contrapunto respecto a los alcances de los enfoques cualitativos, menos hegemónicos, como el de la presente propuesta. Asimismo, los estudios de corte fenomenológico de Lolich^(28)^ en Argentina, enriquecen las perspectivas con otras vertientes cualitativas, como lo es el uso del paradigma interpretativo bajo los principios de la teoría fundamentada en el relato de eventos. Todavía hay una interesante línea de trabajo relativamente más reciente, que ha incursionado en la exploración de los canales digitales y redes sociales informatizadas^(29)^, siendo un hecho que la población adulta, lejos de permanecer estática ante los avances de la modernidad, está cada vez más permeable ante la tecnología y los diferentes recursos digitales como medios emergentes en la expresión de la identidad.

Finalmente, se reconoce que entablar una relación comunicativa de calidez y cercanía, genera un vínculo terapéutico sólido, que facilita el profundizar en las dimensiones que afectan al individuo, lo que redunda como un factor de adherencia para dicho vínculo, permitiendo así gestionar de mejor modo herramientas que impacten sobre la calidad de vida de las personas. Este estudio presenta limitaciones relacionadas con imbricarse con mayor profundidad en la valoración del bienestar subjetivo involucrado en el acto dialógico de recordar, así como también por no profundizar de un modo más directo en elementos de participación comunitaria y/o propios de la relación con otras dimensiones de interés en salud, como lo es la vivencia de la enfermedad, el duelo o el dolor físico^(30)^. La comunalidad de entrevistas autobiográficas deviene de solicitar narrativas de recuerdos contextualizados por elementos circunstanciales, enfatizando en la calidad de aspectos tales como su valencia emocional, las coordenadas de tiempo - espacio, la perspectiva desde donde se evocan (escenarios, representaciones o la mezcla de ambos), entre otros. Esto plantea un importante desafío para la disciplina de la fonoaudiología, interesada en transitar hacia una valoración de la comunicación como elemento constitutivo e integral del ser humano, reconociendo a la vez lo complejidad de llevar una actividad tan íntima y personal como el rememorar al paradigma que existe detrás de cualquier condición de atención clínica tradicional.

## CONCLUSIÓN

Las entrevistas autobiográficas se sitúan como herramientas de interés para los profesionales que trabajan con personas mayores, dada la versatilidad e integralidad que este instrumento otorga a la exploración; su uso en clínica puede verse limitado entre otros por falta de conocimiento, aprehensiones respecto a su extensión o por temor a verse sobrepasado por información, que no al no está tradicionalmente orientada hacia los impedimentos y dishabilidades pueden hacer que el clínico prefiera desestimarlas. En este caso, las facilitaciones otorgadas por el tenor conversacional de una entrevista con orientación autobiográfica favorecen la naturalidad del relato personal, el énfasis especifico que cada uno les da a los hechos/acontecimientos, con lo que los fenómenos cobran vigencia, interés y valor de un modo único, situación que los procedimientos tradicionales enmascaran, pues suelen ser conducidos bajo un formato de interrogación.

No obstante, hay profesionales en la Fonoaudiología que han sido lo suficientemente visionarios para construir conocimiento disciplinar basado en los diferentes aportes que las concepciones de funcionalidad, bienestar y participación han significado para los beneficiarios, a la par de una fuerte incorporación de una práctica cada vez más basada en la evidencia. Este promisorio escenario permite que la Entrevista Autobiográfica sea una alternativa que habilite competencias clínicas altamente deseables como son la capacidad de autorreflexión y empatía en los clínicos, así como permitirle a nuestros beneficiarios una intervención lingüístico - comunicativa verdaderamente integral, incorporando a la tradición de práctica, la preocupación de ofrecer un andamiaje para la autodeterminación de la identidad, el empoderamiento colectivo, la búsqueda de generatividad, trascendencia y propósito de vida en la población etaria más envejecida.
